# Combined Dynamic Light Scattering and Raman Spectroscopy Approach for Characterizing the Aggregation of Therapeutic Proteins

**DOI:** 10.3390/molecules191220888

**Published:** 2014-12-12

**Authors:** E. Neil Lewis, Wei Qi, Linda H. Kidder, Samiul Amin, Stacy M. Kenyon, Steven Blake

**Affiliations:** Malvern Biosciences Inc., 7221 Lee Deforest Drive, Suite 300, Columbia, MD 21046, USA; E-Mails: wei.qi@malvern.com (W.Q.); linda.kidder@malvern.com (L.H.K.); samiul.amin@malvern.com (S.A.); stacy.kenyon@malvern.com (S.M.K.); steve.blake@malvern.com (S.B.)

**Keywords:** dynamic light scattering, Raman spectroscopy, lysozyme, protein aggregation, self-assembly

## Abstract

Determination of the physicochemical properties of protein therapeutics and their aggregates is critical for developing formulations that enhance product efficacy, stability, safety and manufacturability. Analytical challenges are compounded for materials: (1) that are formulated at high concentration, (2) that are formulated with a variety of excipients, and (3) that are available only in small volumes. In this article, a new instrument is described that measures protein secondary and tertiary structure, as well as molecular size, over a range of concentrations and formulation conditions of low volume samples. Specifically, characterization of colloidal and conformational stability is obtained through a combination of two well-established analytical techniques: dynamic light scattering (DLS) and Raman spectroscopy, respectively. As the data for these two analytical modalities are collected on the same sample at the same time, the technique enables direct correlation between them, in addition to the more straightforward benefit of minimizing sample usage by providing multiple analytical measurements on the same aliquot non-destructively. The ability to differentiate between unfolding and aggregation that the combination of these techniques provides enables insights into underlying protein aggregation mechanisms. The article will report on mechanistic insights for aggregation that have been obtained from the application of this technique to the characterization of lysozyme, which was evaluated as a function of concentration and pH.

## 1. Introduction

The focus of development in the pharmaceutical industry has, in recent years, seen a significant shift towards protein-based therapeutics, with the promise of safer, more efficacious products that have fewer side effects. The industry is targeting formulations that allow for subcutaneous delivery that can be self-administered, rather than those that require intravenous delivery in a health clinic or hospital. This goal imposes several constraints: low volume (<1.5 mL), low viscosity (~<10 cP), and sufficient bioavailability to minimize the number of required injections per day [1]. Towards that end, high concentration drug products, often greater than 100 mg/mL, are becoming standard. At these levels, self-association and self-assembly of protein molecules becomes more likely, leading to high viscosity and the formation of aggregates [2,3,4,5].

The degree of reversibility of aggregates that may form is an important characteristic of a specific formulation, and contributes to the understanding of the root causes of instability. Transient or weak interactions result in the formation of oligomers that form and interchange dynamically, and can usually be reversed upon dilution or change of pH of the surrounding environment [6,7,8]. Other aggregates however, are formed as a result of secondary and tertiary structural changes induced in the constituent proteins, in which the interaction of the unfolded (or structurally disturbed) proteins is so strong that they are essentially “irreversible” on times scales and concentration ranges of practical interest [9].

As the formulation instabilities induced by high concentrations are caused by colloidal as well as conformational effects, being able to characterize the changes that occur during aggregation at the micro- and meso-scale, as well as at the molecular structural/conformational scale, is essential to develop in-depth product understanding. Successful characterization therefore, may require multiple techniques to probe these multiple scales. In this study, we report a unique combination of dynamic light scattering (DLS) and Raman spectroscopy to provide novel mechanistic insights into protein aggregation by characterizing these changes, with the goal to guide formulation conditions that maximize stability by targeting the specific root cause.

Although there are a number of instruments which have traditionally been utilized to characterize molecular level structure and conformation in aggregating proteins, many of these suffer limitations. Circular dichroism (CD) and fluorescence (both intrinsic and extrinsic), for example, require low concentration; and as a result, samples that have been diluted for measurement purposes may have different stability characteristics than the high concentrations at which they are formulated, and information obtained may not be directly relevant. Another workhorse characterization technique, DSC is not always able to determine the onset of soluble aggregate formation [10]. Interference from buffer components is another common limitation, and requires that samples be prepared differently for analysis. Particularly for Fourier Transform Infrared (FTIR) spectroscopy, interference from water presents a significant analytical challenge. Also, although FTIR is capable of providing secondary structural information of high concentration samples, it is not sensitive to amino acid side chain environments that enable tertiary structure to be monitored, and cannot characterize size changes [11,12].

Raman spectroscopy has been used for decades in academic environments to characterize secondary and tertiary structure, as well as disulfide bond conformations of proteins [13,14]. Its commercial application has been relatively limited until recently, when advances in detector, laser and fiber-optic technology aligned to enable greatly improved overall sensitivity. State of the art Raman spectroscopy overcomes many of the traditional limitations enumerated above for the study of high concentration formulations: (1) the higher the concentration of the protein, the better the Raman signal; (2) spectral contributions particularly from water are weak and relatively straightforward to subtract; (3) secondary structure (amide I, III), tertiary structure (aromatic side chains—tryptophan and tyrosine) and disulfide conformation markers are all available from a single measurement. The addition of DLS is expected to further expand the breadth of knowledge by providing concurrent sizing information, which enables the correlation of these changes with protein structure. As the same sample is used for both measurements, the variability inherent in performing the measurements separately is minimized. More importantly for therapeutic proteins, the combination can provide a stability profile encompassing both conformational and colloidal stability as the protein is subjected to various stresses,* i.e.*, thermal, chemical, denaturants or time.

**Figure 1:**
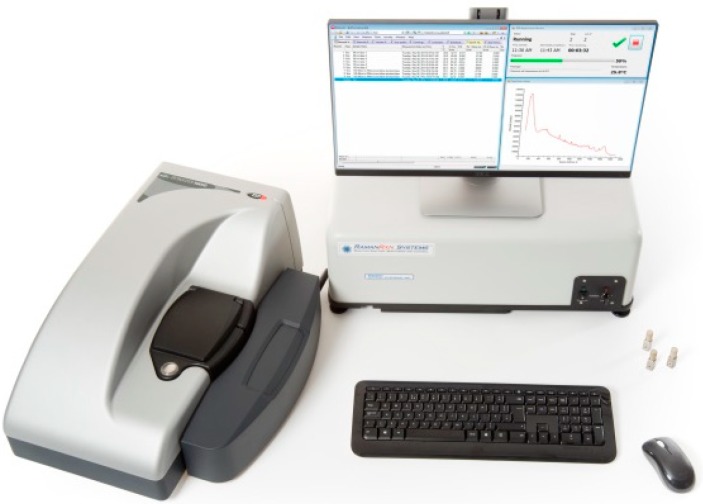
Single instrument platform that combines dynamic light scattering with Raman spectroscopy.

This study exemplifies the utility of a combined DLS and Raman spectroscopy approach through a detailed investigation of lysozyme formulated under different pH and concentrations, and subjected to thermal stress. Although lysozyme is a well investigated system, most aggregation studies involving CD and DSC have focused on low concentrations [15] which makes it difficult to factor in effects of aggregation. The combination of DLS and Raman spectroscopy (instrument shown in Figure 1) provides complementary information about both aggregation (size) and unfolding (secondary and tertiary structure), which allows the user to determine the degree of influence from aggregation on the reversibility of protein unfolding and refolding [16].

## 2. Results and Discussion

### 2.1. Lysozyme Thermal Reversibility at pH 4.0—Temperature Jump Experiments

**Figure 2:**
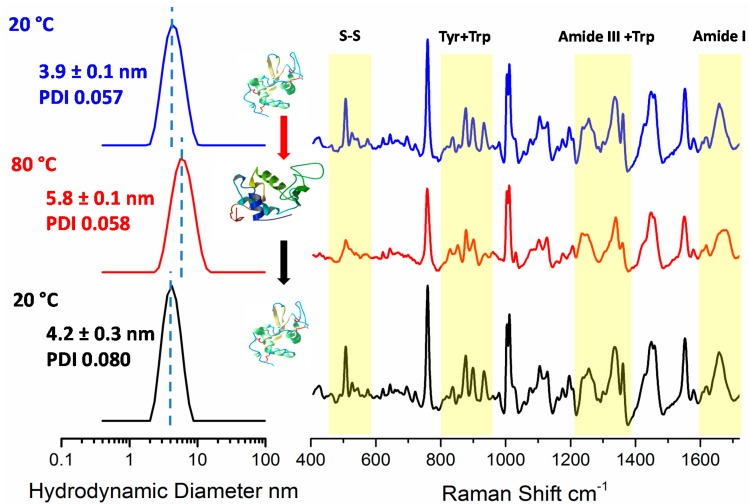
(Left Panel) Representative DLS size and polydispersity measurements of lysozyme at 20 °C (blue), 80 °C (red) and returned to 20 °C (black). (Right Panel) Corresponding Raman spectra. The yellow bands highlight the specific spectral regions discussed in the text. The inserted ribbon models [17] illustrate the three dimensional structure of the folded and unfolded protein.

DLS and Raman spectroscopy was used to probe the unfolding and refolding behavior of a 30 mg/mL lysozyme solution at pH 4.0. This pH was chosen to ensure reversibility of the unfolding/refolding process in the absence of aggregation. The process was initiated by thermal stress, by heating it from 20–80–20 °C while collecting Raman and DLS data (in an interleaved fashion) at these three temperatures. Figure 2 shows DLS and Raman data recorded for lysozyme at 20 °C (blue), 80 °C (red) and on return to 20 °C (black).

The left panel shows the Z-average size of lysozyme at 20 °C (3.9 ± 0.1 nm with a polydispersity index (PDI) of 0.057), at 80 °C (5.8 ± 0.1 nm with a PDI 0.058) and on return to 20 °C (4.2 ± 0.3 nm with a PDI 0.080). Because the PDI is mostly unchanged on heating, the increase in the hydrodynamic size at 80 °C is interpreted as unfolding rather than aggregation, or alternatively the formation of oligomers. As the pI of lysozyme is 11.35, the sample in a pH 4 buffer is highly charged, aggregation is expected to be limited. This further supports the interpretation that the thermal size change is based on unfolding, not aggregation. The final size and PDI values for the sample returned to 20 °C however, are slightly larger than observed at the start of the measurements, which indicates the possibility of limited aggregation or oligomer formation during the cooling cycle. However, the mass percentage of aggregation represented in this distribution is estimated to be less than 0.2%. Given the high protein concentration and the general susceptibility of denatured proteins to aggregate, lysozyme might be expected to display some degree of limited aggregation, even while highly charged in a pH 4 buffer.

In the right panel of Figure 2, we show the corresponding Raman spectra. Measurable changes at each temperature (highlighted in yellow) can be observed that correspond to amide I (1580 to 1720 cm^−1^), amide III (1250–1330 cm^−1^), tryptophan and tyrosine side chains (Trp and Tyr from 800–900 cm^−1^) and disulfide bond conformation (500–700 cm^−1^). These specific changes, and references to their assignments, are detailed in Table 1.

**Table 1:** Changes in the Raman spectral markers during heating to 80 °C [18,19,20,21,22].

Region (Marker at 20 °C)	Change at 80 °C	Implication
Amide III (1257 cm^−1^)	Shifts to 1250 cm^−1^	Loss of α-helix
Amide III (1280 cm^−1^)	Decreases intensity	Loss of α-helix
Amide III (935 cm^−1^)	Increases intensity	Loss of α-helix
Tryptophan (I_1360_/I_1340_)	Band ratio decreases 0.8→0.5	Trp more accessible to aqueous environment
Tyrosine (I_850_/I_830_)	Band ratio increases 0.6→1.7	Tyr more accessible to aqueous environment
Disulfide *g-g-g* conformer (507 cm^−1^)	Decreases intensity with broadening	Loss of vibrational mode
Disulfide *g-g-t* conformer (540 cm^−1^)	Absent	Loss of vibrational mode
Disulfide *t-g-t* conformer (649 cm^−1^)	Absent	Loss of vibrational mode
Amide I ( α-helix content)	43%→27%	Loss of α-helix
Amide I (β-sheet content)	7%→14%	Gain of β-sheet
Amide I (β-turn)	7% (no change)	N/A
Amide I (Random coil content)	27%→37%	Increased random coil
Amide I (1658 cm^−1^)	Broadens	Loss of α-helix; gain of β-sheet/random coil
Amide I (1660 cm^−1^)	Appears	Loss of α-helix; gain of β-sheet/random coil
Amide I (1680 cm^−1^)	Appears	Loss of α-helix; gain of β-sheet/random coil

The key features that emerge from these temperature jump experiments is that the hydrophobic groups become more exposed to an aqueous environment and there is a significant loss of α-helix and gain of β-sheet structure on heating from 20 °C to 80 °C.

### 2.2. Lysozyme Thermal Reversibility at pH 4.0—Temperature Ramp Experiments

To further probe the details of the transition process, a temperature ramp was performed, heating a 30 mg/mL lysozyme sample in pH 4 buffer from 20–80–20 °C, collecting Raman and DLS data (in an interleaved fashion) at 1 °C increments. As each DLS and Raman experiment take ~3 min to collect, the kinetics of this experiment are quite different,* i.e.*, slower, than the temperature jump experiment described in the previous section.

**Figure 3:**
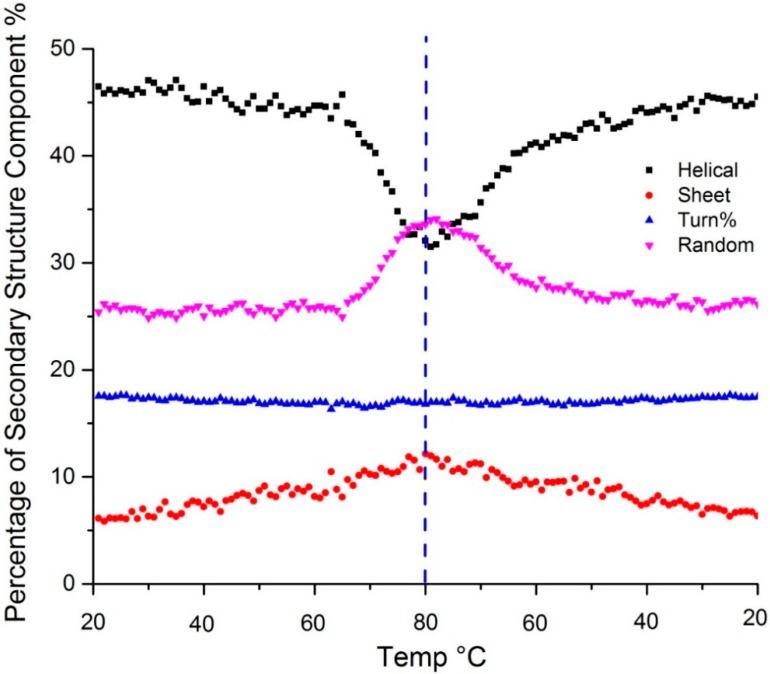
Secondary structure predicted using a PLS model shows change in α-helix (black), β-sheet (red), β-turn (blue) and random coil (purple) content as a function of temperature. The dotted vertical line indicates the point of the highest sample temperature, the point at which the sample ramp changed from heating to cooling.

As may be seen from Table 1 there are many Raman bands whose intensities and frequencies reflect the overall secondary structure of the protein and, as a result a secondary structural analysis is best performed using linear combinations of these changes. Techniques generally referred to as multivariate analysis are used for this purpose [23]. Training data comprised of spectra recorded by the same instrument from a series of proteins with known secondary structures are used to construct a proprietary partial-least squares (PLS) numerical model which is then subsequently employed to automatically predict the secondary structure of unknown protein samples. For the sample at 20 °C the application of the model predicts ~43% α-helical, ~7% β-sheet, ~17% β-turn and ~27% random coil fractions. These values are consistent with the literature values: PDB 1DPX with 41% helical and 10% β-sheet content [17]. At 80 °C, helix content decreases to ~27%, random coil increases significantly to ~37% and β-sheet increases slightly to ~14%. Figure 3 shows the temperature dependent secondary structure content as predicted by the PLS model. Changes in the amide III region all confirm the loss of α-helical structure upon heating: the peak at 1257 shifts to 1250 cm^−1^; the peak at ~1280 cm^−1^ decreases in intensity; the peak intensity at 935 cm^−1^ (C-C skeletal stretching) decreases significantly.

Changes to the tertiary structure are elucidated through a variety of tryptophan and tyrosine markers, many of which have well defined physical interpretations. The I_1360_/I_1340_ peak ratio is a measure of hydrophobicity and associated with a change in the tertiary structure specific to tryptophan side chains [18]. From 20–80 °C, the reported ratio of these markers decreases from 0.8 to 0.5, indicating not only that the local solvent environment of tryptophan has become more hydrophilic but also that the number of tryptophan residues exposed to the aqueous solvent has increased, indicative of unfolding. The peaks at 850 and 830 cm^−1^ are related to the hydrogen bonding environment of the tyrosine side chain. At 20 °C, the 830 cm^−1^ peak is prominent with a shoulder at 850 cm^−1^, but after heating to 80 °C, the 830 cm^−1^ shifts to lower frequency and a distinct peak, in contrast to what was previously a shoulder, appears at 850 cm^−1^. The (I_850_/I_830_) ratio changes from 0.6 to 1.7 on heating, indicating that tyrosine side chains are more accessible to the aqueous environment, also consistent with protein unfolding [21].

The disulfide region of the protein spectrum contains three peaks at 507, 525 and 540 cm^−1^ which represent different conformational isomers: *gauche-gauche-gauche, gauche-gauche-trans* and *trans-gauche-trans* respectively. A C-S stretching peak appears at 690 cm^−1^. With application of thermal stress, the distribution of the conformations change and the relative intensity of these peaks also change: the band intensity at 507 cm^−1^ is greatly reduced; the peak at 540 cm^−1^ broadens and disappears; and the intensity of the C-S stretch at 690 cm^−1^ decreases. It should be noted that these changes are consistent with a change in the conformation of disulfide bonds, not their cleavage. These conformational changes as indicated by the Raman markers are consistent with the loss of tertiary structure and unfolding [20].

The enthalpy of unfolding can be derived by fitting the transition width of a Raman structural parameter plotted as a function of temperature. This value is called the van’t Hoff enthalpy (ΔH_vH_) after the van’t Hoff equation, which gives the temperature dependence of any equilibrium constant. This value differs from the calorimetric enthalpy which is directly calculated from the area under the peak determined via DSC [24]. The van’t Hoff enthalpy assumes a two-state model, whereas the calculation of the calorimetric enthalpy is model-independent. Therefore, the agreement between these two values can be used to confirm two-state behavior [25]. The ΔH_vH_ value obtained from fitting the temperature dependent value of α-helical content is 503.1 ± 32.7 kJ/mol, which compares quite well with calorimetric enthalpy values (~510 kJ/mol) reported for lysozyme samples in pH 4 buffer, even though the DSC samples were run at significantly lower concentrations [26]. The similarity between calorimetric and van’t Hoff enthalpy values confirms the reversibility/two-state behavior of the lysozyme transition.

**Figure 4:**
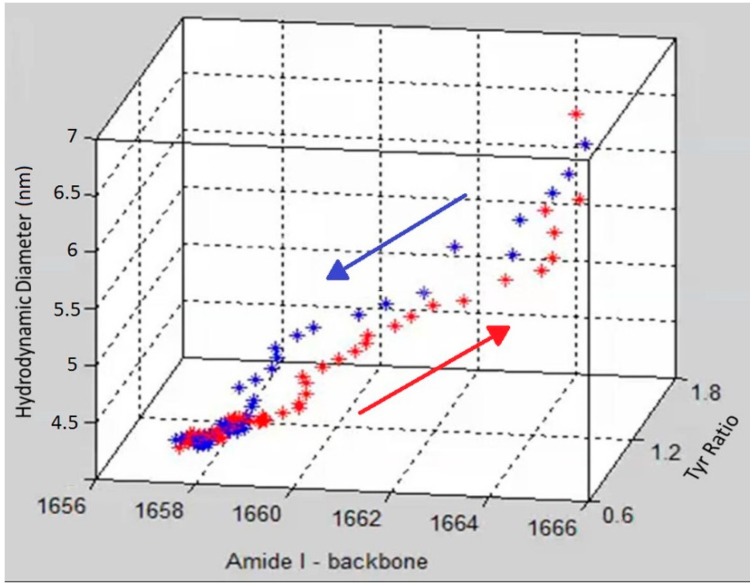
A 3D scatter plot of the trajectory of the unfolding (red) and refolding (blue) of lysozyme at pH 4 as a function of three parameters (size, tertiary and secondary structure).

To demonstrate the complementary nature and correlation between size, secondary and tertiary structure, Figure 4 shows a graph that includes DLS size trends, as well as secondary and tertiary structure trends derived from the Raman data. Specifically, the amide I peak position, hydrodynamic diameter and Tyr I_850_/I_830_ ratio are displayed in Figure 4 as a three dimensional scatter plot, with red points showing the data points from heating and blue points corresponding to the cooling cycle. The initial and final points of the heating and cooling curves overlap for all three parameters but the unfolding and refolding follow different paths. At the start of the heating cycle, the amide I peak position centered at ~1657 cm^−1^ shifts slightly (~2 cm^−1^), during which no corresponding change of any significance in size or tertiary structure is observed. After this initial period of little overall size or structural difference, the unfolding enters a phase of rapid change in tertiary structure as monitored by the tyrosine peak ratio, which changes from 1.5 to ~2. Following this tertiary structure change, the secondary structure, monitored by amide I peak position, under goes significant change, ~5 cm^−1^, as the protein obtains its final unfolded conformation. This data suggests that the change in the tertiary structure occurs before significant change is seen in either secondary structure or size.

### 2.3. Comparison to FTIR—Lysozyme 200 mg/mL pH 4

Raman and FTIR spectroscopy are complementary in that they both measure vibrational frequencies of amino acid functional groups in proteins, but the information is derived from different interactions of electromagnetic radiation with the molecule. As a result the vibrational modes have different selection rules and non-equivalent relative intensities, and in many cases bands that are strong in the Raman spectrum are weak in the infrared and *vice versa*. This contributes to the following well documented differences between the two: (1) Raman is much less sensitive to water, which is critical for simple reproducible measurement of aqueous solutions; (2) Raman is much more sensitive to aromatic side chain vibrations, and therefore provides tertiary structural information in addition to secondary structure; (3) the Raman bands assigned to the S-S stretching vibrations of the protein disulfide bonds are relatively strong but are not observed with FTIR [12].

**Figure 5:**
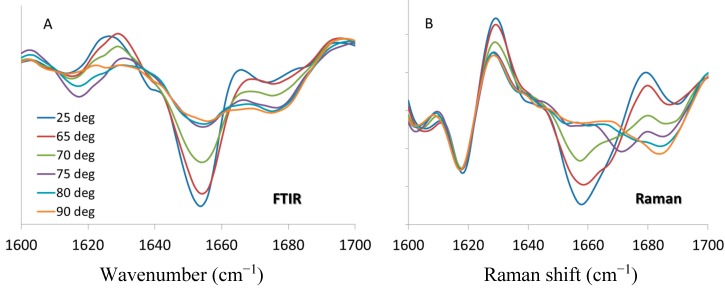
Second derivative spectra of lysozyme at pH 4, 200 mg/mL in the amide I region from 1600 to 1700 cm^−1^ at denoted temperatures. (**A**) FTIR data [12] (**B**) Raman spectra.

Because FTIR is well established as a secondary structure predictor for biopharmaceuticals [27,28,29], here we compare our Raman data to FTIR of lysozyme collected under similar conditions [12]. FTIR (Figure 5A) and Raman (Figure 5B) spectra (with second derivative pre-processing) at the same temperatures are selected, and the results presented for comparison. It should be noted that band positions for certain protein markers may differ,* i.e.*, amide I for FTIR is centered at ~1654 cm^−1^ while for Raman it is at ~1658 cm^−1^. FTIR is routinely used to monitor the formation of inter-molecular beta sheet structure with markers at ~1620 and 1690 cm^−1^ [30,31]; whereas recent studies of insulin and lysozyme amyloid fibril indicate that intermolecular β-sheet structure has a Raman marker at ~1670 cm^−1^ [32,33]. However, the general trend of spectral changes with respect to temperature are consistent and comparable: α-helix content decreases with the application of thermal stress until 75 °C, at which point it remains unchanged with further increase of temperature. For the Raman data, the fitted transition midpoint based on α-helix content is 69.5 ± 0.2 °C, which compares quite well with the FTIR value of 69.8 ± 0.4 °C. The intermolecular β-sheet behavior is a bit more complex. The FTIR spectra in Figure 5A shows a transient increase in the 1620 cm^−1^ marker at 75 and 80 °C which reverses by the time the sample reaches 90°C, with no change exhibited for the 1690 cm^−1^ marker. As shown in Figure 5B the behavior of this marker is similar for the Raman data, showing a temporary increase at 70 and 75 °C. For both the FTIR and Raman, these features are only elevated for two temperature points in the series, reverting to lower values once above the transition temperature. As both the FTIR and Raman spectra show an increase in β-sheet (intramolecular) and β-turn structures at the highest temperatures, bands at ~1680 cm^−1^ (FTIR) and 1685 cm^−1^ (Raman), it is possible that these structures supplant the intermolecular β-sheet that is formed around the transition temperature.

### 2.4. Concentration Effects

DLS and Raman data of six different samples spanning the range 5–120 mg/mL were collected across a heat/cool cycle from 20–80–20 °C, to reveal the impact of concentration on thermal stability and reversibility, from both a colloidal and molecular structural stability perspective. The data were collected at 1 °C increments. Transition midpoint temperatures, derived by plotting helical content as a function of temperature, decrease slightly as concentration increases, from 74.0 ± 0.8 °C to 72.1 ± 0.1 °C, as detailed in Table 2.

**Table 2:** Transition midpoint (T_m_) and corresponding van’t Hoff (ΔH_vH_) enthalpy values derived from fitting the temperature dependence of helical fraction data upon heating, for lysozyme in pH 4.0 buffer at specified concentrations.

Concentration (mg/mL)	T_m_ (°C)	ΔH_vH_ kJ/mol)
5	74.0 ± 0.8	N.A.
10	73.6 ± 0.4	N.A.
20	73.5 ± 0.2	530 ± 79.3
40	73.2 ± 0.2	509 ± 46.7
80	72.7 ± 0.2	529 ±35.8
120	72.1 ± 0.1	568 ± 29.7

**Figure 6:**
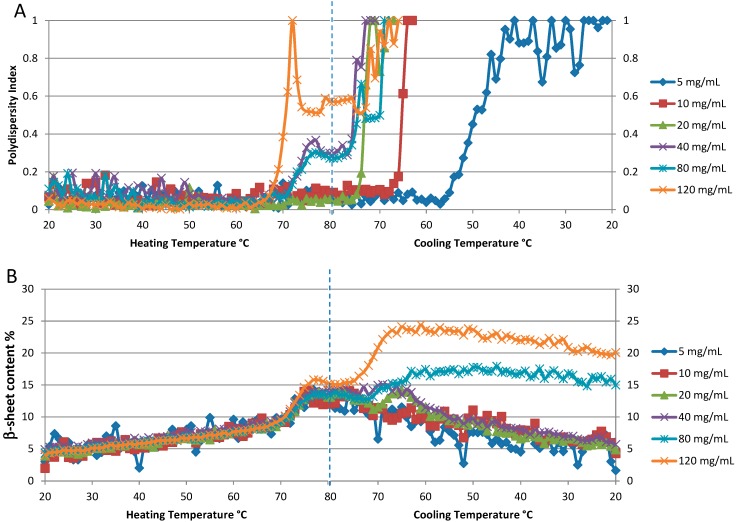
Concentration dependence of thermal reversibility of lysozyme at pH 4: 5, 10, 20, 40, 80 and 120 mg/mL. (**A**). The polydispersity index from DLS data. (**B**). Percentage of β-sheet content derived from Raman spectra.

These T_m_ values are lower than those recorded for samples in similar buffers and at comparable concentrations measured by DSC [26]. In contrast however, as mentioned in the previous section, the T_m_ value reported for a 200 mg/mL solution compares well to the value derived by FTIR [12]. The van’t Hoff enthalpy values for all but the two lowest concentrations are also presented in Table 2. These values are consistent with values determined from DSC measurements, ~510 kJ/mol [26]. Figure 6A shows the polydispersity index (PDI) for the six different concentrations plotted as a function of temperature. Even a qualitative evaluation of the resulting T_onset_ values indicates that aggregation (colloidal stability) is highly dependent on sample concentration. At the three lowest concentrations (5, 10 and 20 mg/mL), the PDI remains below 0.1 through the heating cycle, with no sign of significant increase in this value. However, all three samples exhibit a polydispersity transition to higher values during the cooling cycle: 20 mg/mL is the first to exhibit this behavior with an onset during cooling of ~75 °C, the 10 mg/mL sample follows at ~65 °C, with the lowest concentration sample, 5 mg/mL, being the last to exhibit an increase in polydispersity at ~55 °C. For the three higher concentration samples (40, 80, and 120 mg/mL), the PDI increases during heating. The onset temperature for these PDI transitions is close to 65 °C (on heating) for all three samples, with the most notable difference being that the PDI value increases more sharply for the highest concentration sample, 120 mg/mL.

To aid in interpreting these results, it is important to understand that the PDI value is a measure of inhomogeneous size distribution, and does not necessarily indicate that all of a sample is highly aggregated. As an example of this, the PDI value for the 5 mg/mL sample at the end of the complete heat/cool cycle is 1, the highest possible value; however, the particle size distribution consists of two peaks, with 99.9% of the mass accounted for by the monomer particle population centered at diameter 4.1 nm. Only 0.1% of the sample mass exists as an aggregate, which appears as a single population, centered at ~1.2 μm. The 80 mg/mL sample presents a different story, in that ~100% of the sample weight is accounted for by large aggregates when the PDI value is only 0.5 (during the cooling cycle, ~69 °C). The particle size distribution shows two large populations indicating that severe aggregation has occurred: ~139.4 nm diameter (10.2% wt) and 742.8 nm diameter (89.8% wt). After this temperature, the DLS correlogram cannot be fit with validity, as the number fluctuations induced by large aggregates skew the curve so significantly.

Conformational (spectral) reversibility characteristics are acquired at the same time, and can be examined in conjunction with the colloidal stability information for a more complete story of the sample behavior on heating. As shown in Figure 6B the temperature dependent behavior of β-sheet content is similar for the four lowest concentration samples (5, 10, 20, and 40 mg/mL). Starting at ~5%, this value steadily increases from 20–70 °C, with a period of rapid change from ~70–80 °C, with a maximum value of ~15%. As the samples are cooled, the initial reduction in β-sheet content is slight, but becomes more rapid as the temperature continues to decrease, and eventually returning to within a couple of percentage points of where it started, slightly greater than 5%. This is indicative of a slow but almost complete refolding process. The behavior of this parameter for the two highest concentration samples, 80 and 120 mg/mL, is markedly different, in that the formation of β-sheet content continues even as the sample cools, peaking at 17% and 25%, respectively, before slowly decreasing, but not returning to the original values.

For all concentrations evaluated, lysozyme shows secondary structural change on heating, indicative of partial unfolding. It is the behavior on the subsequent cooling step that exhibits notable concentration-dependent variation. If we consider that unfolding and refolding are competitive processes, and that unfolded molecules can either refold or aggregate, a lysozyme molecule has several possible paths to follow on cooling from an 80 °C temperature maximum. Unfolded species can interact and aggregate, or refold; and previously folded species can unfold. Also, there is a continuum of “in-between” states in which the molecule can be partially folded and/or aggregated. The data presented here support the following concentration-dependent mechanisms: at low concentrations (<80 mg/mL), refolding is more likely than either aggregation or additional unfolding, but at increased concentrations there is a much higher chance of unfolded protein molecules (with exposed hydrophobic areas) interacting and aggregating, than there is of refolding.

The capability to probe this behavior in high concentration, formulated samples can be useful to understand* in situ* therapeutic protein stability. As stated previously, most conventional techniques that probe conformation and stability require low concentration samples, so products designed to be delivered at high concentration have to be diluted to be measured. Therefore, the information gleaned isn’t directly applicable to the sample in its intended marketable form. The best outcome is that the data at different conditions can be used to correlate or predict behavior at formulated conditions. The results presented here demonstrate that thermal stability behavior at low concentration cannot necessarily be extrapolated to higher values, even for a relatively “simple” protein. Additionally, if not accompanied by data that characterizes both conformational and colloidal parameters, the root cause of instability might remain unexplained. Predictive parameters that can be determined at low concentrations, such as k_d_ or B_22_, have been successfully applied [34]. That said, validation of the protein stability predicted by these parameters, both colloidal and conformational, should be verified and validated with samples in their intended formulation conditions. In addition, case studies have shown that for certain cases, k_d_ and B_22_ might *not* be predictive of the sample stability, but rather only of short range interactions [35]. Characterizing protein samples under formulation conditions can help remove the need to make predictive assumptions.

### 2.5. pH Effects

**Figure 7:**
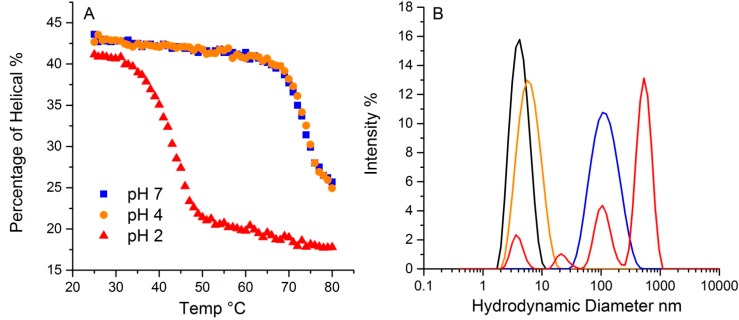
The effect of pH on lysozyme thermal stability revealed by DLS-Raman. (**A**). The percentage of helical along temperature ramp at pH 2, 4 and 7. (**B**). Particle size distribution from dynamic light scattering of lysozyme at pH 2, 4 and 7. The black trace indicates the initial distribution for all three pH values. The colored traces show the resulting size distributions at 80 °C: pH 2 graphed in red, pH 4 in orange, and pH 7 in blue.

The pH in which a protein is solvated plays a crucial role in determining protein conformation and stability. To demonstrate the pH effect on its thermal stability, lysozyme was tested at three different pH values, 2, 4 and 7. As shown in Figure 7A, lysozyme at 20 °C has ~43% helical structure for both pH 4 and 7, with a slightly lower value (~41%) for pH 2. The hydrodynamic radius for all three at 20 °C is ~4.0 nm. Upon thermal ramping, plots of the helical content as a function of temperature for both pH4 and 7 samples almost overlap, with a T_m_ close to 73.4 °C, indicating similar behavior. In contrast, the T_m_ for the sample at pH 2 was 41.2 °C, much lower than the other two samples. Additionally, the helical content that was ultimately reached was also much lower, ~17%, compared to ~24% for the other two pH solutions. This data points to the pH 2 sample having much lower conformational stability. Interestingly, conformational stability does not directly correlate with colloid stability for this sample. For lysozyme at pH 2, multiple peaks appear in the size distribution data, with the largest ~ 500 nm. The mass percentage of such aggregates, however, only account for ~0.3% of the total mass. The samples at pH 4 and 7 have almost indistinguishable conformational stability profiles, but the corresponding hydrodynamic size values, as an indication of a propensity to aggregate and therefore colloidal stability, are very different. At 80 °C, the pH 4 sample shows only a very slight increase in size, which this study has attributed to either unfolding, and/or formation of dimers/trimers. The pH 7 sample, in contrast, does indicate aggregation, with a single peak at much larger size, ~100 nm. In contrast to the pH 2 sample, where only a small fraction of the total mass was accounted for in the aggregate population, there is a complete conversion of the monomer to a size population centered slight above ~100 nm in size. Thus, though their initial percentages of secondary structure are highly similar, and the T_m_ based on the loss of helical structure for samples at pH 4 and 7 are almost identical, the colloidal and conformational stability profiles are vastly different amongst these three samples.

The differences observed here could be attributable to differences in the aggregation mechanisms observed under different pH conditions. The isoelectric point, pI, for lysozyme is around 11.35, so under very acidic conditions, such as pH 2, the monomers are highly charged and the aggregation mechanism under those conditions could be either chain polymerization or nucleation dominated growth. However as the size shows the evolution of larger size aggregates (up to 500 nm) and high polydispersity over a wide range of sizes, chain polymerization seems to be the likely mechanism. This would result in the formation of fibrillar aggregates under these conditions. Chain polymerization seen in other protein systems such as IgG and α-chrymotrypsinogen has also resulted in significantly lower conformational stability [9,10,36]. Under higher pH conditions, at pH 4, the size change shifts to slightly higher values. This would be seen under conditions where dimers and trimers would be formed as a result of nucleation dominated growth. Nucleation dominated growth is usually accompanied by a relatively large loss of conformational stability, similar to that associated with chain polymerization. This, however, is not seen here and in addition, the size shift is very small. This could indicate either the protein is unfolding and there is no aggregation or there is limited aggregation to dimers taking place. The high conformational stability at pH 7 and the shift to larger size aggregates is indicative of cluster-cluster aggregation under these conditions. This again has been observed for a number of different protein systems [9,10]. It should be mentioned that almost all studies on aggregation mechanisms have been carried out at lower concentrations (e.g. 1 mg/mL). The concentration effect on the various aggregation mechanisms has not been investigated in detail and the shift in behavior at pH 4 could be the result of yet another mechanism taking place, whereby either there is only unfolding taking place or small aggregates such as dimers are forming without very high loss in conformational stability. This behavior at pH 4 for very high concentration (200 mg/mL) lysozyme has been observed before through use of FTIR [12], and as has been shown here, concentration can have significant effect on both colloidal and conformational stability. A detailed understanding of the aggregation mechanism and the effect of concentration on those mechanisms is beyond the scope of this article, but will be the focus of future studies. The combination of the sizing and polydispersity data with the structural data from Raman provides a very powerful tool to investigate and characterize the different aggregation mechanisms seen under various formulation conditions.

## 3. Experimental Section

Hen egg white lysozyme (L6876) was purchased from Sigma (St. Louis, MO, USA). Samples were prepared using 20 mM citrate-PBS buffer at specified pH values and concentrations. All the samples were filtered through a 0.22 μm syringe filter from Millipore (Darmstadt, Germany) before any experiments. Concentrations were determined by UV/Vis spectroscopy (NanoDrop 2000) from Thermo Scientific (Wilmington, DE, USA). Data were collected using a Zetasizer Helix instrument (Malvern Instruments Ltd., Malvern, UK), that combines DLS and Raman. The system employs a 785 nm laser (~280 mW) for Raman excitation and a 633 nm laser for the DLS measurements. Approximately 50 μL of sample was introduced to the instrument using a cuvette specifically developed for these integrated measurements. The cell has a thin quartz (125 μm) window to reduce its contribution to the Raman spectra. Thermal ramping data was acquired sequentially from 20 to 80 °C with 1 °C per step unless otherwise specified. The samples were equilibrated for 60 s before each measurement. For Raman collection, typically 15 s accumulation and 10 co-adds were used. For DLS backscattering at 173 °C was collected using an automatically optimized attenuator. For size determination, the temperature dependent viscosity of PBS buffer without protein was used instead of the actual protein solution viscosity value. This implies that the size values for highly viscous samples may not be absolute, but qualitative trends should still be relatively robust. Raman spectra of the buffer were collected over the same temperature range so its contribution could be subtracted from the protein solution spectra to reveal the “protein-only” Raman spectra. Buffer subtraction was effected using an automatic scaling algorithm, and band intensities in the remaining protein spectra were normalized to the phenylalanine peak at 1004 cm^−1^. This peak is assigned to the aromatic ring breathing mode, and its intensity has been shown to remain largely unchanged upon heating and also throughout protein conformational changes. The second derivative spectra were calculated using a standard Savitzky-Golay routine (3rd order fitting and 41 point filter length).

Although the quantitation of secondary structure may be derived from the amide I profile with band fitting, the fitting process requires user input that can make the results subjective, and not entirely reproducible. In addition, this method utilizes only the amide I region, without taking account the wealth of spectral information available in other regions. For this study, a Raman protein library from 18 commonly used model proteins was collected and secondary structure percentages were predicted based on a partial least squares fit of the Raman data to X-ray data reported in the protein structural data base [17].

## 4. Conclusions

In this study we have used the combination of DLS and Raman spectroscopy to derive insights into the mechanistic nature of protein aggregation. Raman is a structural determination technique that overcomes limitations commonly associate with measuring this characteristic—*i.e.*, samples can be evaluated at formulation conditions, rather than being diluted or having to exclude specific interfering excipients. The spectra provide a wealth of secondary and tertiary markers that are specific to particular amino acid side chains and disulfide bonds. As DLS data is collected at the same time, particle size distribution, polydispersity and other related parameters can be combined with the structural data to provide insights into the root cause of formulation instability.

The results determined for this combined method compare well to those from the currently used analytical workhorse instrumentation. Secondary structural data from high concentration samples correlate well with FTIR values, and transition enthalpy values derived from this parameter compare well with DSC results. This study focused on a model protein, lysozyme, and the information obtained was very detailed, providing insights on colloidal and conformational stability, as well as the mechanism of aggregation. It is expected that the correlations between the different types of data (size and structure) are also applicable to mAb-based biotherapeutics and a variety of other types of proteins.

Specifically, from a study of thermal stress of lysozyme, it was determined that: (1) the tertiary structure undergoes a transition earlier than the secondary structure; (2) higher concentrations promote aggregation during cooling that correlates with an irreversible increase of β-sheet structure; and (3) each of three pH values studied yields a unique aggregation pathway, having different conformational and colloidal stability profiles associated with them. This detailed analysis leads to the overall conclusion that colloidal and conformational stability are not directly correlated under many condition-dependent circumstances. It follows therefore, that in order to determine the root cause of formulation instability, a technique that characterizes both of these aspects must be applied. Also clear is that such a system should be able to measure high concentration, formulated samples directly in order to predict behavior under the conditions that matters most—the fully formulated drug product.
